# Ecology of *Ahasverus advena* in Stored Products and Other Habitats

**DOI:** 10.3390/insects16030313

**Published:** 2025-03-18

**Authors:** David W. Hagstrum, Bhadriraju Subramanyam

**Affiliations:** 1 Department of Entomology, Kansas State University, Manhattan, KS 66506, USA; 2 Department of Grain Science and Industry, Kansas State University, Manhattan, KS 66506, USA

**Keywords:** distribution, seasonality, commodities infested, facilities infested, economic losses, extension, quarantine, pest management

## Abstract

The foreign grain beetle, *Ahasverus advena* (Waltl) (Coleoptera: Silvanidae), has been reported from 110 countries on more than 162 commodities, more than 35 types of facilities, and 14 other habitats such as compost heaps and haystacks or manure. Compost heaps, haystacks, and manure heated by fermentation may allow overwintering in cold climates, making them important sources of infestation. From these sources the *A. advena* can fly and infest grain storage and processing facilities. *A. advena* has been found in empty grain storage bins, is often found in wheat immediately after harvest, and is most abundant early in wheat storage. Larvae and adults of *A. advena* are well adapted to feeding on several species of fungi and have higher chitinase levels and greater tolerance for fungal aflatoxins than other species. *A. advena* lay more eggs on the fungal species on which their offspring can develop most successfully. They are attracted to fungal odors and high moisture commodities and have the capability to disseminate grain fungi that cause hot spots within the grain mass. The presence of fungus beetles is indicative of poor storage conditions. *A. advena* is capable of feeding on some commodities and is a predator that may have a potential role in biological control. They are strong fliers but are distributed extensively with the movement of commodities in the marketing system. In countries with a zero tolerance for insects, their presence is sufficient for rejection of a load and associated economic losses. In other countries, contamination by *A. advena* is a problem, and in India, it is listed as a quarantine pest. Extension agents have had many requests for the identification of this species, and two other species of the same genus have been found in stored products. Some information is available for the effectiveness of nine pest management methods for *A. advena*.

## Simple Summary

Extensive published information on geographical distribution, commodities or facilities infested, adaptation to feeding on fungi, and preferences and requirement for high moisture habitats for the foreign grain beetle, *Ahasverus advena* (Waltl), are reviewed in this paper. Life history, population ecology, seasonality, economic losses, and pest management of this pest are discussed, including the spread of *A. advena* by commerce and dispersion by flight. The monitoring of this pest and mention in extension bulletins/factsheets are described. These aspects suggest that *A. advena* can be considered an economically important species, and therefore, greater emphasis should be given to its pest management. This review should encourage further studies on *A. advena* to help us design better pest management and quarantine programs.

## 1. Introduction

The foreign grain beetle, *Ahasverus advena* (Waltl) (Coleoptera: Silvanidae), is a cosmopolitan pest of stored products and an urban pest of apartments and homes in addition to many other habitats. This species was named when it was found among fungi brought to Germany from South America [1]. Goxis [2] said as he moved *Cathartus advena* to a new genus “I will name *Ahasverus*, as much to recall its successive moves as by allusion to the cosmopolitanism”. *Ahasver* is a literary symbol for mobility in general [3] and *advena* may be short for adventive meaning not native to a place or fully established there, but locally or temporarily naturalized. The consensus seems to be that *A. advena* is of American origin [4,5,6,7,8,9,10,11,12] and is now cosmopolitan. Table 1 shows 110 geographic distribution records, 162 commodities infestation records, and 35 facility infestation records for *A. advena*. Early infestation records and dispersal by commerce may be important in explaining current host range and geographic distribution. Biodiversity survey distribution maps are available for *A. advena* in Australia [13], England [14], France [15], and throughout the world [6] (photos of *A. advena* are available at [6]).

## 2. Adaptation to Feeding on Fungi

*A. advena* are well adapted to feeding on several species of fungi with larvae being able to develop on fungi alone. These larvae and adults have higher chitinase levels and greater tolerance for fungal aflatoxins. Adults lay more eggs on the fungal species on which their offspring can develop most successfully. *A. advena* are attracted to fungal odors and high moisture commodities. They are capable of disseminating grain fungi within the grain mass, which cause hot spots. That *A. advena* larvae can develop on fungi alone is evident from fungi grown on chips of porous pot instead of agar being suitable [50] and from agar control without fungi not being suitable in earlier studies [51,52,53,54]. Many of the fungal species investigated are suitable for development and reproduction of *A. advena* (18 out of 43), and eight of these produce toxins (Table 2), although in some cases development was prolonged and reproduction was reduced. Another six fungal species have not been shown to be suitable for either development or reproduction, and five of these produce toxins. *A. advena* larvae or adults were two or three orders of magnitude less sensitive to aflatoxin B1 than other insect species [55]. *A. advena* survived and reproduced better on aflatoxin-producing fungi than the flat grain beetle, *Cryptolestes pusillus* (Schönherr) [52].

Larvae and adults of *A. advena* have 3-fold higher chitinase level than the sawtoothed grain beetle, *Oryzaephilus surinamensis* (L.), giving them a greater ability to utilize fungi [57]. Female *A. advena* laid more eggs on the fungal species on which larval development was successful [51,52]. Six species of soil-borne or seed-borne bacteria diet alone were not suitable for *A. advena* survival [58]. *A. advena* had the lowest threshold for positive response to fungal volatiles at the 10 ng concentration of racemic 3-octanol and was positively attracted over the largest range of concentration of l000-fold compared with three other species [59]. Positive responses to the alcohols and ketones in this study suggest that some of these volatiles might be used as host-finding kairomones in nature. Hot spots in grain caused by fungi and insects are often infested by *A. advena* [60]. *A. advena* is capable of disseminating grain fungi that cause hot spots to healthy grain [61]. Half of *A. advena* and the confused flour beetle, *Tribolium confusum* Jacquelin du Val, migrated to artificially created hot spots immediately after release in shelled corn.

## 3. Preferences and Moisture Requirements

In the laboratory, the percentage of flying *A. advena* attracted to a trap increased 2-fold when it was filled with kibbled wheat, 5-fold with moist pad and 7-fold with both [62]. Odor from wheat stored in bunkers in Australia attracted *A. advena* and five other species associated with moist grain from some distance away [40]. More *A. advena* were captured in ventilator traps in bins with 13% moisture content wheat (520 adults) than bins with 11% moisture content wheat (106 adults) [63]. Wall traps baited with boiled wheat collected almost exclusively *A. advena* and the hairy fungus beetle, *Typhaea stercorea* (L.) in poultry houses [64]. No *A. advena* larvae developed to the adult stage at 58% relative humidity [65].

Adults of *A. advena* are attracted to damp grain and feed on developing fungi [66]. They attack ripening grain in the field in the southern United States and are common in farm stored grain that is beginning to go out of condition. In the United States (Texas, Oklahoma, Kansas and Missouri), *A. advena* was the most abundant species in wheat heads collected from fields (27 out of 150) prior to harvest and particularly on the tips of ears of corn where kernels were rotting [67]. *A. advena* is a strong flyer and can easily locate moldy grain in bins and in fields from far away [68]. In stored oats in Florida, the highest population levels of *A. advena* were concentrated along the wall of the bin with the highest moisture contents [69]. In New Jersey, both *A. advena* and *T. stercorea* were very abundant in 1956 but less so in 1957 when the grain was much drier [70]. This species can overwinter in damp moldy grain in temperate climates of Western Canada, Minnesota, North Dakota and other parts of North America [71].

During the third month of storage in Apapa, Nigeria, the numbers of *A. advena* in the outer region of a bag stack increased to seven adults per groundnut sample as the center of stack dried as result of moisture translocation (center dried to 2% and bottom increased to 8% becoming moldy, caked and rancid as the center heated to 40 °C) [72]. By the middle of the fifth month, *A. advena* was practically extinct even in the outer region. In South Sulawesi, Indonesia, kilogram cocoa bean samples at a trader had *A. advena* in 9.5% of samples and at an exporter had *A. advena* in 33.3% of samples during February, which was a wet season, but none during July, which was a dry season [43]. The most common species initially in Kumasi, Ghana (mean 0.31 per bag in September and 0.52 in October), was *A. advena*; their occurrence is very irregular and suggests a great sensitivity to moisture content [73].

Structural infestations *A. advena* develop in wall voids, crawl spaces, or attics of homes where high humidity supports fungal growth and can be associated with plumbing leaks, condensation problems or poor ventilation [45,74,75,76]. This species can develop on fungus growing on pallets made of new green lumber [77].

## 4. Other Habitats

Habitats other than well-managed stored commodities include new apartments [38,78] or houses [78,79,80,81,82,83], bird nests [84,85,86,87], cattle-grazed grassland [88], compost [17,21,81,89], dregs of pressed grapes [23], fungus produced hot spots in stored grain [90,91], haystacks [81,92,93,94,95,96,97], moldy plant debris [14,98], pallets made of new green lumber [77], poultry manure [48,64,99,100], pearl millet in dough stage in field [101], wood trash during winter [102], stable manure [103], under tree bark [17,104,105] and wasp nests [106]. On farms, *A. advena* may develop in manure heaps and moldy straw bales. They may overwinter in these habitats which are relatively warm and fly to grain stores as the weather warms up [107]. Manure and straw habitats may be potential sources of *A. advena* infestation on farms and should be located well away from stored grain. Literally tens of thousands of *A. advena* were found in haystacks from a very large field miles away from any granaries or warehouses [93]. Large numbers of *A. advena* were found at the bottom of a haystack [94]. A single *A. advena* adult was found during excavation of a ca. 1860 privy or outhouse in Quebec, Canada [108]. *A. advena* could have been attracted to the moist environment of the privy or to fungal odors from the privy or could have been introduced by dumping kitchen trash into the privy.

## 5. Dispersal by Commerce

The dispersal of *A. advena* by commerce is evident from records of their interception in exports (six examples) and imports (nine examples) even though they may be occasionally overlooked because of their small adult length [109,110]), with estimates ranging from 1.2–2.75 mm [37], 1.36–2.23 mm [30], 1.8–2.0 mm [54], 1.8–2.4 mm [111], 1.9–2.5 mm [112], and 2–2.3 mm [7] to 2–3 mm [71,113,114,115].

This species was the insect pest most frequently found in cacao in 60 kg jute bags at warehouses located on the pier of the ports of Ilheus and Bahia, Brazil, with average densities as high as 80–90 insects per bag [116]. In Nigerian transit shed storing palm kernels and palm kernel meal, sticky traps caught 647 [117] and 1206 [49] adults of *A. advena*. In Nigerian ports, *A. advena* spread to groundnuts awaiting shipment [118]. Sticky traps caught 298 *A. advena* adults over an 8-day period in cocoa palm kernel store in Lagos, Nigeria. This species was found in cocoa, groundnut or ground nut cake cargos of three of five ships when cargo was inspected in transit sheds on ships in Apapa, Nigeria, and produce and ship upon its arrival in the UK [32]. *A. advena* was detected frequently only on groundnuts on one ship in Apapa and not in the transit shed or upon ship arrival in the UK. This species was detected very frequently on cocoa in transit sheds, and upon the arrival of a second ship in the UK, but not on a ship in Apapa. A third ship had *A. advena* infestation detected only upon its arrival in the UK, frequently in cocoa and very frequently in groundnut cake. Numbers of *A. advena* captured in suction traps operated between 16.00 and 21.00 h every fourth night in a shed at Ikeja, Nigeria, with a 1250-ton stack of bagged cocoa were 53 in the first trapping in February and 1 to 9 in subsequent trappings [119]. The bag stack was studied during the dry season, the breakdown and exportation occurred before the heavy rains began and the moisture content of cocoa remained constant at 6.6%.

In Belgium, a total of 6882 *A. advena* were found in 229 cocoa bean samples imported between 1991 and 1996, decreasing from 85 to 58% of all insects in the samples [120]. For cocoa beans imported into Belgium, *A. advena* was 81% of insects from South American, 61% of insects from African and 39% of insects from Far-East Asian countries [121]. This species was detected in 31% of 665 cargos of cocao beans, 16% of 1257 cargos of palm kernels, 11% of 539 cargos of decorticated peanuts and 3% of 156 cargos of in-shell peanuts imported into England from Africa [122]. They were also found in 81 out of 339 cargos of other commodities. They were numerous but unevenly distributed. From Orient, Sago, flour (6 cargos) was infested by *A. advena*, and Illipenuts (27 cargos) were more frequently infested by *A. advena* [123]. The number of *A. advena*-infested cargos tended to decrease from 1957 to 1969. Copra (44 cargos) and cocoa beans (22 cargos) from Ethiopia, Orient and Pacific Islands were infested by *A. advena*. Also, palm kernels (13 cargos) from Ethiopia and the Orient and grain (6 cargos) from the Orient had *A. advena* infestation. This species infested 0.9% of oilseed and 0.8% of cereal and cereal product cargos imported into Japan from South East Asia from 1955 to 1957 [124]. In India, *A. advena* has been intercepted 131 times on imported cashews (Table 3).

In the United States, totals were 52 *A. advena* from 24 Chinese cargos, 1 from 1 Malaysian cargo, 2 from 2 Philippine cargos, 3 from 2 South Korean cargos, 1 from 1 Taiwanese cargo, and 2 from 2 Vietnamese cargos [125]. Most of the adults were observed crawling on pallets and shipping container floors. The cargoes were ceramic, hardware and machinery, so *A. advena* must have been from an infestation of wood pallets or residues from previous food cargo. Both *A. advena* and *T. stercorea* were found in 0.1% corn (average density 13.7 per kilogram) exported from the United States, but not wheat exports [126]. Ten commodities imported from nine countries were infested by *A. advena* and with some commodities infested by this species on more than one occasion [127]. There were 19 interceptions between 30 October 1978 and 30 September 1979. Twenty-four commodities imported from nine countries were infested by *A. advena* and with some commodities infested by this species on more than one occasion [128]. There were 61 interceptions from December 1984 to December 1987.

The dispersal of *A. advena* by commerce has been described as follows: “This species [*A. advena*] is carried around the world in association with stored products and was probably first introduced to the Old World by man” [111], “This species [*A. advena*] lives in rice and was first spread over northern Europe with it” and “…should be considered European because it will undoubtedly reproduce in Europe and thus become naturalized” [129] and “For over a century and a half, the species [*A. advena*] has acclimatized in Europe and should be treated as a permanent component of our fauna; apart from synanthropic sites, it also occurs in many natural microbiotopes” [8].

## 6. Flight

The minimal temperature for flight of *A. advena* and *T. stercorea* is 17.5 °C, and the optimal temperatures are 25–27.5 °C, with 82% of *A. advena* and 70% of *T. stercorea* flying at optimal temperatures [130]. Two parasitoids had the same minimal temperature for flight and 100% flight at 25 °C. Three pyralid moths had a lower minimal temperature threshold for flight (12–15 °C), and the lower threshold temperatures for the lesser grain borer, *Rhyzopertha dominica* (F.) and the red flour beetle, *Tribolium castaneum* (Herbst) flight were the same as the optimum for *A. advena*. For a strain of *A. advena* cultured in the laboratory for over 20 years, only a single *A advena* flew at the minimum temperature for flight of 17.5 °C [62]. The minimum temperature for flight initiation by the rusty grain beetle *Cryptolestes ferrugineus* (Stephens) was 20 °C; there was little difference between strains in their low temperature flight thresholds for *A. advena* and *C. ferrugineus* but differences between strains in flight initiation were greater at the near optimum temperature of 25 °C. Low temperature walking thresholds are 10 °C for larvae and 7.5 °C for adult *A. advena* [131]. Adult *A. advena* are excellent climbers of glass [59].

There is one report that *A. advena* does not fly [8]. This species has been caught outdoors in flight traps in Canada [132], Czech Republic [133], England [134], Pakistan [135], Poland [136], and the United States [63,137,138]. This species comprised 1.9% (*n* = 22) of all insects caught in light traps 45 km from Faisalabad, Pakistan [134]. In Kansas, from July to October 2007 and 2008, outdoor corrugated traps at one of three food processing facilities caught more *A. advena* than indoor traps (45 and 26 outdoor vs. 0 and 1 indoor) and outdoor in commercially available Lindgren funnel traps at all three plants from August to September sometimes caught even more *A. advena* (10–95) [138].

In Canada, four species of mites are phoretic on *A. advena* [139]. Phoretic deutonymphs of *Digamasellus punctum* (Berlese) and hypopi of *Acalvolia* sp. near squamata were found under the wings of *A. advena* adults. *D. punctum* and *A.* near *squamata* have been found in stable manure and house dust, respectively. Phoretic female *Dolychocybe* sp. near *indica* were under wings of *A. advena* and phoretic female *Tarsonemus ascitus* Delfinado was on the prothorax of *A. advena*.

## 7. Monitoring

Traps baited with pheromones or food odors are often used for insect monitoring. Individuals of both sexes of *A. advena* produce an aggregation pheromone, 1-octen-3-ol, beginning at four to six weeks posteclosion, which attracts both sexes to infested food [140]. At low densities, pheromone production rates were at least four times greater for males than for females. Pheromone production is suppressed at high *A. advena* densities preventing overcrowding. Odor from wheat stored in bunkers in Australia attracted *A. advena* and five other species associated with moist grain from some distance away catching mean numbers of 0.12 to 1.12 adults per trap in 12.5 to 75% of eight traps per bunker (Barrer 1983) [40].

*A. advena* has a large electroantennographic response to carob volatiles [141]. Pitfall bioassay showed a 77.3% response to 2-nonanone from carob, 78.5% response to 2-undecanone from carob and 84.2% to carob. The Y-tube olfactometer was not suitable for *A. advena* due to the failure of this species to move upwind. This species was trapped in rice (264 or 61.7% of all insect species), maize (122 or 11.83% of all insects) and soybeans (11 or 7.91% of all insects) by food baits in Vietnamese maize granaries [44]. Near-infrared spectroscopy correctly classed 80% of *A. advena* as secondary pests rather than a primary pest [142], an important step toward automating insect identification.

## 8. Population Ecology

Studies in Canadian, Greek, Indonesian and Vietnamese grain storage, on Mexican farms, in American peanut shelling plant, and in coffee storage in Ivory Coast have shown that *A. advena* is often abundant and widely distributed. In three hot spots in wheat stored in Canada, 2–52 adult and 2–48 larval *A. advena* were found, and there were 22 adults and 17 larval *A. advena* in wheat surrounding the hot spots [90]. In four hot spots in oats, 2–69 adult and 2–57 larval *A. advena* were found, and there were 8 adults and 4 larval *A. advena* in oats surrounding the hot spots. In Canada, *A. advena* is described as the most common fungus beetle found in the Prairie Provinces [143]. Also in Canada, *A. advena* was widespread and found twelve times in elevators, nine times in flour mills and once at a feed mill in 2465 grain inspection samples from 24 cities [71]. Over 9200 *C. ferrugineus* and *A. advena* were caught in probe trap place near hot spots [144]. This species was found in 13 out of 444 grain samples of 1000 to 1500 mL each in Manitoba, 22 out of 762 in Saskatchewan and 1 out of 546 in Alberta between mid-June and mid-August 1981, 1982 and 1984 [145]. Adults of *A. advena* were found in grain residues in 21% of empty granaries with a maximum of three insects per granary, and these small densities may contribute to carrying over the population when new grain is stored [146].

A mean of 40 *A. advena* adults were caught in brown rice bait bags on top of four rice bag stacks, 82 on the side and 15 on the floor in Indonesia over seven months [39]. In Vietnam, many *A. advena* (264 or 61.7% of all insect species) were trapped with long-grain rice food bait from March-May 2019 inside maize granaries at Mai Son district and Son La province [44]. Totals of 53 and 166 *A. advena* were captured in dome traps inside two peanut shelling plants in the southeastern United States [137]. Flight traps captured one adult inside and six adults outside one of the shelling plants. Totals of 25 and 55 *A. advena* were collected in food bait traps located near farm houses, sheds storing equipment or housing goats, sheep, horses or poultry on 20.4% of 44 farms in Nuevo Leon, Mexico, and 30.1% of 53 farms in Tamaulipas, Mexico [147]. In Ivory Coast, *A. advena* (24.4% all insects) was the second most abundant insect infesting coffee beans next to *T. castaneum* (38.4%) [20]. Damage (4.4%) by the dried fruit beetle, *Carpophilus hemipterus* (L.) was beneficial to *A. advena*, allowing them to consume coffee beans.

In the United States (Florida, Kansas, Kentucky, Minnesota, Oklahoma, South Carolina, South Dakota, Texas, Wisconsin and 18 other states), *A. advena* was often abundant and widely distributed in stored grain. In stored oats in Florida, the highest population levels of *A. advena* were concentrated along the wall of the bin coinciding roughly with the highest moisture contents [69].

In stored wheat in Kansas, *A. advena* was more evenly distributed among three regions (headspace, surface and grain mass) of a bin than the other species [148]. The estimated mean total number of *A. advena* caught in ventilation traps as they enter bins was 5.8 per day (Hagstrum 2001) [149]. The mean number of *A. advena* captured in ventilation traps at the bin cap was 15 times higher than those captured in ventilation traps at the bin eaves. This may be a result of hot air in the bin headspace rising and carrying an odor plume out the cap to attract *A. advena* so that they are more likely to enter at the cap. During the fourth week of storage, probe traps captured a mean of 3.5 *A. advena* in 32 out of 34 bins. Traps near the bin wall captured 3.8- to 5.8-fold fewer *A. advena* than those in the center. This species was found in both traps and grain samples in 57.1% of 14 bins of newly harvested wheat on eight farms in Kansas and traps in all of the bins [150]. More *A. advena* were trapped in the center of the bin (88.8 adults) than against the bin wall (6.3 adults). Traps inserted with the top below the surface of the grain collected an average of 4.1 times more adults than those with the top above the surface [151]. Traps detected *A. advena* 37 days before they were detected in grain samples. Fewer *A. advena* adults were found in traps or grain samples after 90 days of storage when the temperature had decreased to 23 °C. This species was one of the five most common species on farms in Kansas, being found in 23% of 13 corn bins, 23% of 9 oat bins and 19% of 154 wheat bins [152]. The mean number of *A. advena* per kilogram varied among locations in elevators, i.e., boot pit = 0.11, dump pit = 0.02, headhouse = 0.01, rail area = 0.05, and tunnel = 0.43 [153]. This species was caught in pitfall or sticky traps in all 20 feed mills, with 58 in receiving, 629 in mill interior, 12 in load out and 157 on mill exterior [154].

In Kentucky, sampling 134 bins on 114 farms in 24 counties, *A. advena* were found in 25% of bins in 53% of counties in 1989 and 63% of bins in 92% of counties in 1990 [155]. An average of 2.8 *A. advena* adults per 0.3 L of shelled corn were found in the center of the bin and 0.8 *A. advena* adults per 0.3 L of shelled corn near the outside edge of the bin in 1989, and an average of 16.8 *A. advena* adults per 0.3 L of shelled corn were found in the center and 7.2 *A. advena* adults per 0.3 L of shelled corn near the outside edge of the bin in 1990. One to forty-two *A. advena* adults were captured by probe traps in four out of five round bins and two flat units storing shelled corn in Minnesota [156]. In Oklahoma, the mean number of *A. advena* in unbaited traps were 0.42 for flight traps and 6.81 for probe traps and 0.006 in grain samples [157]. The ratio of the number of *A. advena* adults in probe traps compared to the number in grain samples was 1, 293:1. In South Carolina, *A. advena* infestations were found in grain samples from 4.8% of bins of stored corn, 10.6% of bins of stored wheat, 4.1% of empty bins, 7.8% of 51 farms and 8.2% in 46 counties [158]. An average of two *A. advena* adults per 0.9 L grain sample was found at one or two farms in three counties in South Carolina [159]. In South Dakota, *A. advena* was one of the five most common species infesting oats, i.e., infesting 8% of 58 bins with a mean of 19 *A. advena* adults in 1982–1983 and 5% of 21 bins with a mean of 18 *A. advena* adults in 1983–1984 [160]. In both stored rice in Texas (total 420 vs. 30) and stored wheat in Kansas (total 533 vs. 58), more *A. advena* were caught in probe traps in grain than in sticky traps in bin headspace [161]. In Wisconsin, a total of 755 *A*. *advena* were collected in all 20 feed mills in 1975, and a total of 185 *A. advena* were collected in 17 out of 18 feed mills in 1976 by taking a 250 mL sample of whole grain corn, oats, grain dust and spilled feed from each feed mill and trapping eight times biweekly from June to August [162]. On farms in 27 states in the United States, *A. advena* infested 0.2% of wheat with an average density of 4 adults per 1000 g and 16% of corn with an average density of 21 adults per 1000 g, and no *A. advena* were found in oats [163]. For more on the ecology of *A. advena* and some additional references, see [164].

In many studies, large numbers of *A. advena* were observed, i.e., 1206 [49], 1807 [165], 3602 [63], 5278 [148], 5887 [166], 6882 [120], 8757 [165], 13,278 [116]. There were 300 complaints of *A. advena* in apartments in Germany [38]. A total of 1897 occurrences of *A. advena* mainly in outdoors habitats have been reported between 1897 and 2024, many with photographs of adult *A. advena* [6]. A total of 146 occurrences of *A. advena* have been reported for England, with earliest being 1913 and most between 1972 and 2024 [14], and in Australia, 66 occurrences have been reported [13].

## 9. Life History

The development of *A. advena* from oviposition to adult at 27 °C in a rolled oats-yeast medium was 19.1 days in 92% relative humidity, and 24.2 days in 66% relative humidity, where four to five instars were observed [65]. No *A. advena* developed to the adult stage at 58% relative humidity. When fully grown, the larva of *A. advena* stopped feeding, constructed a chamber by cementing food particles together and attaching itself to substrate with a brownish substance secreted from the anal aperture. At 27 °C, the preoviposition period was 3–4 days at constant 75% relative humidity, and the preoviposition period was 4–20 days at constant 58% relative humidity with 65% of the females not ovipositing. On egg-laying days at 75% relative humidity and 27 °C, the number of eggs per female was 1–4 or occasionally 8–12. Each female oviposited for 20–30 days, stopped for 5–23 days, and then resumed egg laying. There were normally 2–4 non-ovipositional periods per female. Average total number of eggs laid over 135 days was 198 (range 107–300) per female. Longevity averaged 159 days for mated males, 208 days for mated females, 275 for unmated males and 301 days for unmated females.

At 70% relative humidity, egg-to-adult development on kibbled wheat took 67, 58 and 48 days at 20, 22.5 and 25 °C, respectively, while corresponding cumulative mortality was 94, 52 and 65% [107]. At 90% relative humidity, oviposition to adult development on kibbled wheat took 70, 46, 31, 26, 21, 16 and 22 days at 17.5, 20, 22.5, 25, 27.5, 30 and 32.5 °C, while corresponding cumulative mortality was 94, 68, 63, 44, 58, 45 and 57%. At 90% relative humidity, the mean longevity increased from 69 days at 10 °C to 267 days at 15’C and then declined to 99 days at 25 °C. Fifty 0–3-day-old adult *A. advena* produced a mean of 125, 216 and 235 progeny in 3 weeks at 90% relative humidity and 17.5, 20 and 25 °C, respectively. Mortality was 97% at 5 °C and 95% at 7.5 °C in 28 days with complete mortality at 5 °C within 20 days, and at 3 °C, it was about 16 days [167]. Endosymbionts were found in *A. advena* [168,169], but infection rates were low or questionable. Adult survival of *A. advena* reared on a mixture of rolled oats and brewer’s yeast powder was adversely affected by higher dockage levels at 30 °C and 33 °C but not at 27 °C, and adverse effects were progressively more pronounced as the dockage was increased [170]. A black mutant of *A. advena* was discovered in 1960 [171].

At 65% but not 60% relative humidity, *A. advena* can develop on a diet of rolled oats or whole wheat flour with wheat germ but not whole kernel wheat [12]. Adding yeast improved breeding success. Damaged copra, cocoa, peanuts and palm kernels were detrimental to all stages of *A. advena* possibly due to oil on the surface. There is photographic evidence that *A. advena* can damage peanuts (p. 13, plate 1) [172]. Autoclaved wheat germ was not a suitable diet for *A. advena* unless a small amount of yeast was added before autoclaving [56]. Living fungi were not essential for development. *A. advena* larvae penetrated corn kernel in the germ area, consuming the entire endosperm before pupating within the kernel [54]. The length of *A. advena* larvae in mm, head capsule width in mm and duration of larval stage in days are 0.81, 0.19 and 3.6 for the first instar; 1.55, 0.26 and 2.4 for the second instar; 2.98, 0.33 and 2.4 for the third instar; 4.0, 0.43 and 2.2 for the fourth instar; and 4.37, 0.45 and 2.2 for the fifth instar [71].

## 10. Seasonality

Occurrences of *A. advena* may appear irregular because adults can fly long distances and easily find moist moldy food or warm places to overwinter. This species may be the first to arrive as the newly harvested commodity is stored and may die out as commodity dries. Much of the data on seasonal variation were collected in North America, but some data are available from Europe, Asia and Africa. In freshly harvested wheat in Kansas [173,174,175] and corn in Kentucky [155,176,177], *A. advena* infestations begin early in storage. *A. advena* caught in sticky traps peaked at >50 per 2 weeks in 1995 and >190 per 2 weeks in 1996 in September, corresponding to the peak in flight activity for this species in Kentucky [176]. The sudden switch in *A. advena* from sticky traps to probe traps between September and October in both years is a shift in activity from the headspace to the grain mass. Outdoors in Kansas, the first flying *A. advena* were trapped from 10 to 16 June 1942 and the last were trapped from 10 to 22 October 1942 [63]. Both *A. advena* and *Ahasverus rectus* (LeConte), a related species, were found in wood trash during the winter in South Carolina [102].

In Illinois, *A. advena* adults comprised 13.7 to 55.9% of all insects in shelled corn samples from aerated bins from August to December of 1958 with the highest percentages in August and October [178]. In non-aerated bins, adult *A. advena* ranged from 6.1 to 10.2% of all insects in November and December of 1957. In Minnesota, *A. advena* was common in 11 wheat bins sampled from May 1977 to October 1978 [179]. For shelled corn, *A. advena* was found in 5.4% of 37 bins in May, 50% of 36 bins in July–August and 52% of 25 bins in September–October. In the winter of 1959–1960 in Canada, *A. advena* was first detected in 150 g samples of stored oats with a hot spot on 22 February (oats 18–38 °C), which increased by 7 March (oats 29–38 °C) and then decreased by 21 March (oats 36–38 °C) [91]. In a second oat bin, *A. advena* was first detected on 7 March (oats 21–29 °C), and similar numbers were found on 11 April (oats 18–21 °C). In Canada, *A. advena* was trapped only in heated granaries in Canada during May–November [180]. This species was caught in probe traps in 9% of farm granaries with a maximum of 7 insects/trap/week in the fall of 1986, in 7% of granaries with a maximum of 1 insect/trap/week in the summer of 1987 and in 46% of granaries with a maximum of 100 insects/trap/week in the fall of 1987 [181]. *A. advena* were more common in granaries with aeration, probably due to odors emanating from the grain during aeration.

Captures of *A. advena* in wheat seed warehouse on unbaited glue boards around the overhead doors peaked in early September 2004 (>45 adults per month) and then tapered off through early November of 2004 [182]. The majority of *A. advena* were captured near the floor (>120 adults per month), and captures decreased with height above the floor. At a flour mill in Kansas, *A. advena* was absent or scarce during the winter months [183]. In Alabama, *A. advena* populations infested stored peanuts with a peak of 134 in November 1953 and then were somewhat consistent from March to August 1954 (20 to 57 per bushel) [184]. Over 45 weeks, most of 8757 *A. advena* individuals were caught in sticky traps above a minimum of 23 °C on three farms in South Carolina. Most were captured between April and late September 1987 [165]. Over 64 weeks, most of 1807 *A. advena* individuals were caught in food bait traps containing corn above a minimum 12.8 °C from late August 1986 to late September 1987 on the same three farms. Sticky traps in ventilators caught the first *A. advena* adult entering bins between 10th and 16th June 1987.

In England, *A. advena* was captured in commercially available Johnson–Taylor insect suction traps (see [132] for the manufacturer’s name) on 11 May 1954 in a field of young winter wheat at Ardington, Berks [134]. In South Sulawesi, Indonesia, *A. advena* were present in cocoa beans at trader and exporter during the February wet season but not during the July dry season [43]. This species was present in maize sold at market by local maize traders between October 2001 and April 2002 in southern Ghana [46]. The number of adult *A. advena* reported outdoors in an 1890 to 2023 diversity survey increased from February to August and then declined until December with another increase in January [6]. Zero to six specimens per month of *A. advena* have been sent to Iowa State University for identification from California, Florida, Iowa, Massachusetts, Michigan, Missouri, New Jersey, New York, Ontario, Tennessee, Texas and Wisconsin during the months of May to November, mostly during June to August [185]. In late August and early September, as weather cools and rainfall increases, *A. advena* adults enter homes as they look for places to spend the winter [77]. Increased numbers of *A. advena* have been observed in Iowa and Indiana [79], Kentucky [83] and Wisconsin [186] in late August and early September.

## 11. Economic Losses and Extension

Required zero tolerance for insects in grain trading and export in the United Kingdom means that even the presence of *A. advena* is often sufficient for the rejection of loads with the consequence of financial losses due to returned loads and their subsequent disinfestation. Everywhere, contamination of commodities with insect bodies, cast skins and frass can be a problem. In India, *A. advena* is said to be absent [10,187], and it is listed as a quarantine pest in schedule VI of PQ Order, 2003, as it is frequently intercepted in cashews imported into India (Table 3), and fumigations and reinspection add to the cost of the imported shipments. In Russia, *A. advena* is a species with a restricted distribution [188]. An infestation of *A. advena* initially misidentified as *T. castaneum* cost a company in excess of USD 2 million to eliminate it [189]. Also, this species may be capable of disseminating grain fungi that cause hot spots [61] and can serve as reservoirs for bacterial diseases [64,190]. The extent to which *A. advena* moves between stored food and natural habitats such as compost, haystacks and manure could make them an important reservoir and vector of microbial disease. *A. advena* can be cannibalistic [8,54], and this could contribute to their vectoring ability by consuming infected insects.

**Table 3:** Raw cashew nuts imported and intercepted in India with *Ahasverus advena* during 2018–2020. Modified from [187].

Country	Total Shipments	MT Imported	Interceptions
Benin	594	46,050	25
Burkina Faso	460	33,287	8
Cote d Ivoire	1664	196,227	20
Gambia	236	16,416	9
Ghana	2013	190,080	21
Guinea	677	60,346	20
Guinea Bissau	813	127,258	5
Indonesia	223	18,581	1
Madagascar	30	1483	2
Mali	9	571	5
Mozambique	167	21,432	1
Nigeria	909	60,950	7
Senegal	306	34,561	3
Tanzania	297	44,385	2
Togo	202	12,428	2
Total	8600	864,055	131

On the American continent, *A. advena* has the greatest economic importance mainly in corn storage, and it can also be a predator with a role in biological control [8]. *A. advena* reduced the coffee berry borer *Hypothenemus hampei* (Ferrari) by up to 70.1% when it was in the tree and by up to 76.4% when it was on the ground [191]. *A. advena* adults killed up to 63.2% of *H. hampei*, and *A. advena* larvae killed up to 42.3% of *H. hampei* in the laboratory [192].

Specimens of *A. advena* have been sent to extension personnel for identification [193,194,195], and this species has been the subject of extension bulletins and pest control industry articles [68,77,196,197]. Although moldy, out-of-condition grain or grain products are the traditional sources of *A. advena*, structural infestations develop in wall voids, crawl spaces, or attics where high humidity supports fungal growth [45,74,75,76,78]. This species feeds on the fungi that grow on wood, lumber, plaster, or wallboards that have not dried completely. Damp wood or accumulations of sawdust behind walls can support fungi that attract the beetles. The insect normally does not develop when the relative humidity is below 65%. Problems with *A. advena* tend to appear in new homes (2 to 3 years old), following renovation of older homes, or in structures with moisture problems. This species can be associated with plumbing leaks, condensation problems, or poor ventilation. Sudden emergences of huge numbers of adult *A. advena* usually become a problem in late summer (around August or early September) when they move out of wall voids and are attracted to fly to windows and lights.

## 12. Pest Management

*A. advena* populations reach high densities during the warmer months, suggesting that more emphasis should be placed on their control [183]. Toews [183] stated that “…outside activity of *A. advena* is less well known and managers have no commercial pheromones or other attractants to monitor outside the mill and make predictions on the potential for immigration.”. Fungal volatiles [59], *A. advena*-produced aggregation pheromone [140] and food attractants from carobs [141] have been identified as potential attractants for monitoring of *A. advena*.

The presence of fungus beetles is indicative of poor storage conditions [111]. Biocontrol by *Xylocoris flavipes* (Reuter) [198], fogging [73,183], heat treatment [199], insecticides [70,167,200,201,202], mass trapping with UV light [100,166], liquid fumigants [70], ozone [203], methyl bromide [183], phosphine [27,69,116,204,205] or sulfuryl fluoride [206] fumigation, sorbic acid [207] and stirring [208] provide effective control of *A. advena*.

In four 500-bushel bins in Georgia, *X. flavipes* reduced *A. advena* by 72.9% [198]. *Cephalonomia waterstoni* Gahan, *C. tarsalis* (Ashmead) and *Xylocoris galactinus* (Fieber) also are natural enemies of *A. advena* [71]. Lower numbers of *A. advena* in the commercial warehouse than the plantation warehouse was attributed to it having more of the predator *Thaneroclerus buquet* (Lefebvre) [47]. Captures of *A. advena* sometimes increased significantly immediately following the August 2004 fogging with dichlorvos + pyrethrin of the warehouse and drying room at a flour mill in Kansas and decreased following the October 2004 methyl bromide fumigation [183]. This species may be susceptible to the oil alone during pyrethrin fogging of cocoa beans in Ghana increasing from <2 to >10 adults per bag from November 1957 to April 1958 and then declining until June 1958 [73]. Heat treatment killed 100% of *A. advena* in a Kansas flour mill during a test in 1999 [199].

Six studies have investigated the effect of 12 insecticides and a mixture of 3 on *A. advena*. Insecticide applications in 1956–1957 substantially reduced *A. advena* populations from 44 insects per 0.45 kg of wheat in control to 0 with malathion and 0, 1.9 or 4.3 per 0.45 kg of wheat with pyrethrins [70]. Pirimiphos-methyl, etrimfos and chlorpyrifos-methyl killed *A. advena* equally well (100% within 2 days at 3–10 °C, with one exception, chlorpyrifos-methyl required 8 days at 3 °C) [167]. In Serbia, populations of *A. advena* on sunflower seed prior to insecticide application were 12.4, 20.4 and 82 per kg and mortality of *A. advena* was 100% with malathion wettable powder, emulsion concentrate and dust formulations [201]. Mortality of *A. advena* was 100% at each concentration of chlorpyrifos-methyl, etrimfos, methacrifos and pirimiphos-methyl [202]. After 1 day, *A. advena* mortality was 100% with pirimiphos-methyl, fenitrothion, chlorpyrifos-methyl, etrimfos, chlorpyrifos, permethrin, deltamethrin, bioallethrin, alphacypermethrin and bendiocarb when applied to wood, steel or concrete, and 100% with mixture of fenitrothion, permethrin and resmethrin when applied to wood or steel, but only 90% when applied to concrete [200]. The label requirements for using an insecticide vary with the country’s regulations and cannot be adequately covered in this review.

The potential for mass trapping of *A. advena* with light traps has been investigated in Indonesian rice warehouses and in a poultry farm in Germany. Wet UV light traps caught 5761 and 5887 *A. advena* individuals in two Indonesian rice warehouses and may be able to help reduce infestations [166]. Traps with ultraviolet lights in poultry house were able to catch such a high proportion of *A. advena* before they laid their eggs (425 adults caught prior to day 10 of trapping, and fewer than 25 adults were caught between day 11 and 24 as estimated from a graph) that the development of a breeding population was largely prevented (only 300 adults caught between day 25 and 36 of trapping as estimated from a graph) [100].

Eight studies have investigated the effect of four fumigants on *A. advena,* and one study investigated the effect of grain stirring on *A. advena*. Liquid fumigant containing ethylene dibromide, ethylene dichloride, carbon bisulphide and carbon tetrachloride applied 6 weeks after harvest killed within a week 97% of the population in which *O. surinamensis*, *A. advena*, *C. ferrugineus* and *T. stercorea* were the most predominant species [70]. The numbers of *A. advena* captured in pitfall probe traps in 8.9 tonnes (350 bushels) of maize were lower in the ozone-treated bin compared with the control bin [203]. Phosphine fumigation reduced *A. advena* populations in bagged cocoa beans [116], and oats stored on farm [69]. In cocoa beans imported from Cameroon, Ivory Coast and Dominican Republic, *A. advena* were susceptible to phosphine [205]. Surviving despite double phosphine fumigation in a feed factory suggested some resistance in *A. advena* [204]. Mortality rates of adult stages in laboratory at 23 °C after 24 h exposure to 20 ppm phosphine was sufficient to completely kill adults. In the Mandalay Region, a dry zone of Myanmar, phosphine fumigation reduced the number of *A. advena* in bags of green pea, cowpea, butterbean and groundnut [27]. For even tolerant species such as *A. advena* at cool temperatures, the length of exposure to sulfuryl fluoride is the key to reducing the dosage levels required to kill eggs to the practically feasible level of 1500 g h/m^3^ [206]. A stirring process reduced the numbers of *A. advena* in stored grain, and none were found after 40 days [208].

Other studies on *A. advena* have considered the effect of increased use of aeration of grain bins or recommend drying corn better, keeping sources of *A. advena* breeding as far away as possible from grain storage, and ultralow dichlorvos fogging instead of dichlorvos strips under plastic sheets for cocoa beans. More extensive use of aeration in Germany has decreased insect infestations in stored grain in February from 11% in 1976 to 1.2% in 1986, but there was a relative increase in importance of *A. advena* infestations [209], perhaps as result of their contribution to producing hot spots. In June 2019, a large infestation by *A. advena* was observed in a mass of corn grains from the city of Plácido de Castro, in the state of Acre, Brazil, for the first time [210]. Proper drying of corn to about 13% moisture content was recommended to reduce risks of *A. advena* infestation. Because compost, haystacks and poultry manure can provide some heat for overwintering in cold climates and be an important source of *A. advena*, keeping them as far away as possible from grain storage can be an important part of pest management [107]. Plastic sheeting is unacceptable for control of *A. advena* infesting cocoa beans in dockside warehouses in Norfolk, Virginia, because this treatment resulted in higher humidities, higher moisture contents and more *A. advena* (20 in covered, no dichlorvos strips, 112 in covered with dichlorvos strips, 13 in uncovered, no dichlorvos strips and 96 in uncovered, dichlorvos strips) [41]. Some warehouses now use an ultralow volume application of dichlorvos with a fogger instead of the strips. Given the ceramic, hardware and machinery cargos in [125] study, the importance of fumigating shipping containers to prevent infestation of a food cargo by *A. advena* is evident.

## 13. Related Species

A closely related species, *A. rectus*, has been found to be associated with stored products [69,211,212,213], has a wide distribution in the United States and may be mistaken for *A. advena* [214]. *A. rectus* has been the most prevalent beetle collected in UV light traps in Florida and has been captured in pitfall traps located in an orange grove. It is abundant in grass clippings, in old squares of sod and in grass tufts around sandy areas. All over Alabama, *A. advena* is present, and *A. rectus* was found in Auburn, Lee County, infesting corn [213]. On Horn Island, a barrier island off the Mississippi coast accessible only by boat, which has been a ranch, military base and currently a wildlife refuge, *A. rectus* has been found [215]. A total of 567 occurrences of *A. rectus* have been reported with the earliest record in 1944 and the rest between 1972 and 2024 mostly from the United States but also 1 from Panama and 25 records from the Bahamas [216]. Keys [217], photographs [65] and drawings [23] are available for identifying larvae and mandibles [218] of *A. advena*. Male and female pupae of *A. advena* can be separated by differences in pupal genital appendages [219].

*Ahasverus excisus* (Reitter), originally described in association with Havana cigars imported to Germany, has widespread distribution in Central and South America (Brazil, Guatemala, Mexico, Panama, Suriname, Trinidad, Venezuela), where it has not been reported in storage habitats (Halstead 1993) [111]. A total of seven occurrences of *A. excisus* have been reported [220].

## 14. Conclusions

The extensive literature on *A. advena* suggests that this species can be economically important and that greater emphasis should be given to its pest management. *A. advena* is particularly a problem, reaching high densities, on cocoa beans, copra, corn, groundnuts, palm kernels, oats, peanuts, rice and wheat, but can be associated with many commodities. It may be a predator of some importance. The breeding of *A. advena* in 14 habitats other than well-managed stored commodities may make pest management of *A. advena* more difficult. The extensive published information on geographical distribution, infested commodities or facilities, adaptation to feeding on fungi, preference and requirement for high moisture, other *A. advena* habitats, spread by commerce and flight, monitoring, distribution, life history, population ecology, seasonality, economic losses, extension, pest management and related species should encourage further studies on *A advena* and help us to design better pest management and quarantine programs. More research is needed on the extent of commodity weight loss due to *A. advena* feeding, particularly on corn and peanuts, their importance as vectors of disease and their importance as predators. Comprehensive studies on the behavior, ecology and pest management of *A. advena* are needed to highlight its status as an important pest of stored products.

## Figures and Tables

**Table 1:** Geographic distribution, commodities infested, and facilities infested by *Ahasverus advena* ^1^.

**Geographic Distribution**
Albania, Algeria, Argentina, Ascension [6], Austria, Azerbaijan [6], Australia, Bangladesh, Belarus, Belgium, Benin (Dahomey was located within present-day Benin from approximately 1600 until 1904) [16], Bosnia Herzegovina, Bulgaria [17], Brazil, Burundi, Cameroon, Canada, Canary Is., Chile, China, Colombia, Congo, Croatia, Cuba, Czech Republic, Denmark, Dominican Republic, Ecuador, England, Estonia [6], Ethiopia, Finland, France, French Guiana [6], Germany, Ghana, Greece, Grenada, Guadeloupe [18], Honduras, Hungary, India, Indonesia, Iran, Ireland [19], Israel, Italy, Ivory Coast (Cote d’Ivoire) [20], Jamaica, Japan, Kyrgyzstan [17], Latvia [17], Lesotho, Liberia, Libya, Leichtenstein, Lithuania, Luxemburg [6], Madeira, Malawi, Malaysia, Mexico, Micronesia, Mona [18], Monaco [6], Morocco, Montenegro [6], Mozambique, the Netherlands [6], New Caledonia, New Zealand, Nigeria, Norway [21], Pakistan, Panama, Papua New Guinea, Paraguay, Philippines, Poland, Portugal, Puerto Rico, Romania, Russia, Rwanda, Samoa, Siberia, Singapore, Slovakia, Slovenia, Solomon Is., South Africa, Spain, Sri Lanka, St Helena [6], St. Lucia, Suriname, Sweden, Switzerland, Taiwan, Thailand, Tonga Trinidad, Tobago, Tunisia [17], Turkey, Uganda, Ukraine [17], the United States, Vietnam
**Commodities**
Algarroba pod, alfalfa green meal [22], almond hull [23], animal feed, apple [24], apple pomace, apricot seed, bamboo [25], barley, BBQ sauce, bean, bean curd, biscuit, black pepper, bone, book [25], Brazil nut (para nut), broom stick [26]), bulb, butterbean [27], cake, cashew, cassava root chip, cassava root meal, castor bean, cereal grain, cereal product, chilli bean sauce, chilli pepper paste, Chinese traditional herbal medicine, chocolate, cinnamon [25], cocoa, cocoa bean, cocoa cake, coffee, coffee bean, confectionary [28], copra, copra meal, coriander, cottonseed, cottonseed meal pellet, cowpea, currants [29], dandelion medicine, date, dried bamboo leaf or shoot, dried banana, dried cassava root (manioc), dried chilli pepper pod, dried chrysanthemum flower (pyrethrum), dried fig, dried flowers [25], dried fruit, dried fungus, dried leaf, dried lily flower, dried mushroom, dried mussel, dried root (tuber), dried seafood, dried shrimp, dried soup mix, dried tomato, durum wheat [30], eggplant, fennel seed, fern [25]), fig, fish, flax, *gugijagung* (maize steamed with cassava and dried) [31]), garlic, ginger, grain product, grass seed, groundnut cake [32]), hay, herb, herbal medicine, illipenut, Indian madder medicine, kidney bean, kiwi, lac [30], licorice apricot, linseed meal, litchi nut (lychee), *Lonchocarpus* root (rotenone), maize, maize bran, maize cob, maize flour [30], malt, mango, medicinal plants [33], melon, melon rind, milled rice [31]), millet, moldy grain, nut, nutmeg (mace), oat, oilcake, oilseed, olive, onion, orange, orange pomace, orchard grass seed, paddy, palm kernel, palm kernel meal, papyrus mats [25], pea [34], peach, peanut (groundnut), peanut oilcake, pecan [35], pigeon pea (arhar, red gram), pine nut, pineapple, plum, prune, pulse, quince [36], raisin, rice, rice meal, root (tuber), safflower, sago flour, sal seed [37], senna pod medicine, sesame seed, sheanut, sorghum (broomcorn, milo), soybean, spice, sunflower seed, sweet potato, tamarind, tapioca, tapioca shred, taro, teak leaf [30], textured soybean flour [34], timber [26], tobacco leaf, tomato, turmeric, turtle shell, vegetable, wheat, wheat bran (pollard), wheat flour, wheat meal, wheat straw [25], wicker basketware, yam, yellow bean sauce
**Facilities**
Apartment [38], bag stack [39], barley mills, bunker storage [40], central storage [39], cocoa storages, commercial godown [31], co-op storage [31], currant raisin storages, dockside warehouse [41], empty cargo containers, estate warehouse [42], exporter [43], farm grain bins, farm storages of rice, feed mills, flat grain storages, flour mills, government godown [31], grain elevators, granary [44], house [45], local maize trader market [46], peanut shelling plants, peanut warehouses, pecan storage [35], pet stores, plantation warehouse [47], poultry house [48], railroad cars, retail stores [31], rice warehouse, semolina mills, sultana raisin storages, transit shed [49], wholesale stores [31]

^1^ Majority of the records are from [7] and new records are followed by cited references.

**Table 2:** Suitability of fungal species for *Ahasverus advena* larval development, adult survival, and reproduction.

Fungus Species	Egg to Adult ^1^	Adult Survival ^1^	Fecundity ^2^	Cited Paper
*Alternaria tenuis* Nees	24 *		yes	[54]
*Aspergillus amstelodami* Thom & Church	17–20		94	[51]
*Aspergillus amstelodami* Thom & Church			32	[52]
*Aspergillus amstelodami* Thom & Church	16			[50]
*Aspergillus candidus* Link	22–34			[51]
*Aspergillus candidus* Link	22–23 *			[54]
*Aspergillus candidus* Link	17			[50]
*Aspergillus candidus* Link	25–54		yes	[54]
*Aspergillus candidus* Link			59	[52]
*Aspergillus chevalieri* Thom & Churck			50	[52]
*Aspergillus chevalieri* Thom & Churck	19			[56]
*Aspergillus chevalieri* Thom & Churck				
var *intermedia* Thom & Raper	15			[50]
*Aspergillus clavatus* Desmazieres		no *		[53]
*Aspergillus conicus* Blochwitz	18			[50]
*Aspergillus flavus* Link	no			[51]
*Aspergillus flavus* Link		yes *		[53]
*Aspergillus flavus* Link	no			[50]
*Aspergillus flavus* Link strain 108.08	no *		no	[54]
*Aspergillus flavus* Link strain 119.62	no *		no	[54]
*Aspergillus flavus* Link strain 242.65	62 *		no	[54]
*Aspergillus flavus* Link			6	[52]
*Aspergillus fumigatus* Fresenius		yes *		[53]
*Aspergillus nidulans* G Winter		no *		[53]
*Aspergillus niger* van Tiegham	no		no	[54]
*Aspergillus niger* van Tiegham	no			[51]
*Aspergillus niger* van Tiegham	no			[50]
*Aspergillus ochraceus* Wilhelm		yes *		[53]
*Aspergillus ochraceus* Wilhelm	no *			[51]
*Aspergillus ochraceus* Wilhelm	no *		no	[54]
*Aspergillus ochraceus* Wilhelm			no	[52]
*Aspergillus oryzae* Cohn	25		yes	[54]
*Aspergillus parasiticus* Speare		yes *		[53]
*Aspergillus parasiticus* Speare	no		no	[54]
*Aspergillus parasiticus* Speare			2	[52]
*Aspergillus repens* Thom & Churk	15			[50]
*Aspergillus repens* Thom & Churk	25–54		yes	[54]
*Aspergillus restrictus* G. Smith	19			[56]
*Aspergillus ruber* Thom & Churk	52		1	[52]
*Aspergillus ruber* Thom & Churk	25*		yes	[54]
*Aspergillus tamari* Kita	no		no	[54]
*Aspergillus terreus* Thom	no		no	[54]
*Aspergillus versicolor* Tiraboschi	25–28 *		yes	[54]
*Aspergillus versicolor* Tiraboschi		yes *		[53]
*Cladosporium herbarum* Link	54		yes	[54]
*Cladosporium* sp.	19–24		110	[51]
*Claviceps purpurea* (Fr.) Tul.		yes *		[53]
*Epicoccum purpurescens* Link	54		yes	[54]
*Fusarium roseum* Link		yes *		[53]
*Fusarium sporotrichioides* Sherb.		no *		[53]
*Fusarium trictnctum* (Corda) Sacc.		yes *		[53]
*Gliocladum fimbriatum* Gilman & Abbott			no	[52]
*Oidium* sp. Lk. Em. Sacc.	54		yes	[54]
*Penicillium album* Sochal	25		yes	[54]
*Penicillium citrinum* Thom	16–23 *		76	[51]
*Penicillium citrinum* Thom NRRL 1842			23	[52]
*Penicillium citrinum* Thom NRRL 805			34	[52]
*Penicillium citrinum* Thom		yes *		[53]
*Penicillium citrinum* Thom	25 *		yes	[54]
*Penicillium claviforme* Bainier		yes		[53]
*Penicillium cyclopium* Sochal	25 *		yes	[54]
*Penicillium fellutenum* Biourge		yes *		[53]
*Penicillium islandicum* Sopp		yes *		[53]
*Penicillium lividum* Westl	no			[54]
*Penicillium oxalicum* Currie & Thom	54		yes	[54]
*Penicillium roquefortii* Thom	54–55 *		yes	[54]
*Penicillium rubrum* O. Stoll		no *		[53]
*Penicillium stoloniferum* Thom	54		yes	[54]
*Penicillium terrestre* Jensen		yes		[53]
*Penicillium urticae* Bainier		yes *		[53]
*Sporendonema baharnensis* C.P.	25		yes	[54]
*Sporendonema brumotii* Salvanet-Duval	24–25		yes	[54]

^1^ In both 1964 [56] and 1978 [50], Hill reported minimum egg-to-adult developmental times. * indicates toxin-producing fungus. ^2^ Rodriguez [52] gave numbers of larvae produced. David and Mills 1975 [51] gave mean number of eggs per 15 days.

## Data Availability

Data sharing is not applicable to this article.
